# Diagnostic accuracy of OCTA and OCT for myopic choroidal neovascularisation: a systematic review and meta-analysis

**DOI:** 10.1038/s41433-022-02227-8

**Published:** 2022-12-02

**Authors:** Sharon Ho, Angelica Ly, Kyoko Ohno-Matsui, Michael Kalloniatis, Gordon S. Doig

**Affiliations:** 1 Centre for Eye Health, UNSW Medicine and Health, University of New South Wales, Sydney, NSW Australia; 2 School of Optometry and Vision Science, UNSW Medicine and Health, University of New South Wales, Sydney, NSW Australia; 3 Brien Holden Vision Institute, University of New South Wales, Sydney, NSW Australia; 4 Department of Ophthalmology and Visual Science, Tokyo Medical and Dental University, Tokyo, Japan

**Keywords:** Retinal diseases, Diagnosis

## Abstract

**Background/Objectives:**

The purpose of this project was to systematically review and meta-analyse studies assessing the diagnostic accuracy of optical coherence tomography angiography (OCTA) and optical coherence tomography (OCT) for myopic choroidal neovascularisation (mCNV). Fluorescein angiography (FA) was accepted as the reference standard.

**Methods:**

PUBMED and EMBASE were searched from inception to March 2021 for studies evaluating the test accuracy of OCTA and/or OCT for diagnosing mCNV. The Preferred Reporting Items for Systematic Reviews and Meta-analyses of Diagnostic Test Accuracy Studies guideline was followed, and the Grading of Recommendations, Assessment, Development and Evaluation approach was used to frame clinical recommendations. Pooled estimates of test accuracy were obtained using a bivariate model.

**Results:**

Of 410 studies assessed for eligibility, 3 studies were identified that compared OCTA to FA and 3 studies were identified that compared spectral domain (SD) OCT to FA. All studies had at least one major methodological flaw leading to an overall high risk of bias. On meta-analysis, the pooled sensitivity of OCTA was 0.89 (95% CI 0.78–0.94) and pooled specificity was 0.93 (95% CI 0.79–0.98). The pooled sensitivity of SD-OCT was 0.99 (95% CI 0.91–1.00). Due to uncertainty in individual studies, the pooled specificity of SD-OCT could not be estimated.

**Conclusions:**

OCTA can reliably diagnose mCNV in clinically suspected patients, however, SD-OCT may not reliably establish a positive diagnosis of mCNV. Future large, prospective studies with improvements in conduct and reporting are needed to strengthen these clinical recommendations.

## Abstract 2

**Learning Objectives:**

Upon completion of this activity, participants will be able to:

1. Describe test accuracy, pooled sensitivity and pooled specificity of optical coherence tomography angiography (OCTA) and spectral domain (SD)-OCT in diagnosing myopic choroidal neovascularization (mCNV) compared with fluorescein angiography (FA) as the reference standard, according to a meta-analysis.
2. Determine clinical recommendations for using OCTA and SD-OCT in diagnosing mCNV, according to a meta-analysis.
3. Identify other clinical and research implications of test accuracy, pooled sensitivity and pooled specificity of OCTA and SD-OCT in diagnosing mCNV compared with FA as the reference standard, according to a meta-analysis.

**Accreditation Statements:**

In support of improving patient care, this activity has been planned and implemented by Medscape, LLC and Springer Nature. Medscape, LLC is jointly accredited with commendation by the Accreditation Council for Continuing Medical Education (ACCME), the Accreditation Council for Pharmacy Education (ACPE), and the American Nurses Credentialing Center (ANCC), to provide continuing education for the healthcare team.

Medscape, LLC designates this Journal-based CME activity for a maximum of 1.0 *AMA PRA Category 1 Credit(s)*™. Physicians should claim only the credit commensurate with the extent of their participation in the activity.

To participate in this journal CME activity: (1) review the learning objectives and author disclosures; (2) study the education content; (3) take the post-test with a 75% minimum passing score and complete the evaluation at http://www.medscape.org/journal/eye; (4) view/print certificate.

**Credit hours:**

1.0

**Release date:**

**Expiration date:**

**Post-test link:** https://medscape.org/eye/posttest978466

**Journal CME author disclosure information:**

Laurie Barclay, MD Freelance writer and reviewer, Medscape, LLC, and has disclosed the following relevant financial relationships: formerly owned stocks in AbbVie.

## Introduction

Approximately 166 million people around the world have myopic macular degeneration [1]. During their lifetime, up to 11.3% of these people will develop sight-threatening myopic choroidal neovascularisation (mCNV) [2]. Due to the progressive natural history of mCNV and time-dependent nature of vision restoration using anti-vascular endothelial growth factor treatments, there is an urgent need to understand how innovative imaging technologies can be used to facilitate early and accurate diagnosis of mCNV [2, 3].

Fluorescein angiography (FA) is the current reference standard for diagnosing mCNV [4]. However, FA is an invasive technique [5] and causes adverse systemic reactions in 4.8% of patients, with life-threatening anaphylaxis occurring in up to 0.3% of all patients [6, 7]. Optical coherence tomography angiography (OCTA) and optical coherence tomography (OCT) represent quicker and safer imaging technologies that do not require systemic dye injection, thus avoiding FA’s associated complications [4]. Whilst multiple comprehensive reviews have described the potential value of using OCTA and/or OCT for the diagnosis of mCNV [4, 5, 8, 9], we are unaware of any studies that have undertaken a meta-analysis to assess overall pooled diagnostic test accuracy.

The primary purpose of this study was to conduct a systematic review and meta-analysis to determine the test accuracy of OCTA and OCT in diagnosing mCNV compared to the reference standard, FA. The primary outcome for meta-analysis was pooled sensitivity and specificity. Pooled likelihood ratios were also estimated, and eligible studies were assessed to determine if direct measures of health consequences (patient-oriented outcomes) could be considered.

## Materials and methods

This systematic review and meta-analysis was conducted and reported in compliance with the Preferred Reporting Items for Systematic Review and Meta-analysis of Diagnostic Test Accuracy Studies (PRISMA-DTA) [10] and the Grading of Recommendations, Assessment, Development and Evaluation (GRADE) guidelines [11, 12]. The study protocol was published online prior to commencing the literature search [13]. Study selection, data extraction and risk of bias appraisal were undertaken by at least two authors (SH, GSD). Disagreements were resolved by obtaining the opinion of a third independent author (AL) and majority decisions prevailed.

### Data sources

MEDLINE (www.PubMed.org) and EMBASE (www.Embase.com) were searched from inception until 30 March 2021, without language restrictions. The query combined appropriate database specific statements using Medical Subject Heading (MeSH) or Emtree terms, and filters for studies of diagnostic accuracy were applied [14–16]. Reference lists of retrieved papers were also hand-searched to identify additional studies. Complete search strategy details are reported in Supplement eTable 1.

### Study selection

We included all studies evaluating the test accuracy of OCTA and/or OCT against the reference standard FA in diagnosing mCNV. Any combination of OCTA and/or OCT test device assessed against FA was included. Exclusion criteria were case reports, review articles, non-human studies and any article type where primary data to calculate sensitivity and specificity against the reference standard was not completely reported.

Endnote software (Clarivate Analytics) was used to manage references. After removal of duplicate studies, articles were screened by title and abstract to identify studies that needed to be retrieved in full text for detailed assessment of eligibility.

### Data extraction and risk of bias assessment

Data regarding study design, population, index test and reference test procedures, and outcomes were extracted from eligible studies. All included studies were appraised for risk of bias using the following domains from the revised Quality Assessment of Diagnostic Accuracy Studies (QUADAS-2) tool: [17] (1) was consecutive or random sampling used to obtain the patient sample; (2) was a case-control design avoided; (3) were there inappropriate exclusions; (4) were index test results interpreted without knowledge of the reference standard results; (5) were reference standard results interpreted without knowledge of the index test results; (6) was there an appropriate interval between the index test and reference standard; and (7) were all patients included in the analysis. Studies were rated ‘high’, ‘low’ or ‘unclear’ for risk of bias, and results graphed using a ‘traffic light system’ as proposed in the GRADE guidelines [11]. The ‘unclear’ category was used only when insufficient data was reported to permit a judgement.

### Data synthesis and analysis

#### Clinical recommendations

The GRADE guidelines for assessing certainty of the evidence with respect to study design, risk of bias, indirectness, inconsistency, imprecision and publication bias were used to frame clinical recommendations [11, 12]. The GRADE assessment framework is supported by published reporting guidelines including PRISMA-DTA and Cochrane [10, 18–20].

### Outcomes

The primary outcome was test accuracy, calculated as the sensitivity and specificity of OCTA or OCT against FA at the initial patient presentation. Downstream consequences of care (management decisions, health outcomes, resource utilisation) [21] delivered using OCTA or OCT alone vs FA were investigated as secondary outcomes.

### Statistical analysis

Given the limitations of all available statistical models and methods to test for publication bias in test accuracy studies, and lack of a standardised method to register test accuracy studies [12], we intended to assess publication bias using Deeks’ test [22] only if 10 or more studies were identified for inclusion.

Pooled estimates with 95% confidence intervals (95% CI) of sensitivity and specificity for each index test (OCTA and OCT) were obtained using a bivariate model. Estimates of the positive likelihood ratio, negative likelihood ratio, and positive and negative predictive values were back-calculated from the pooled estimates of sensitivity and specificity. Study differences in patient populations, patient selection, risk of bias, clinical setting, disease severity, scan density, scan quality and retinal thickness were considered as sources of heterogeneity.

All statistical analyses were conducted using RevMan 5.4.1 (The Cochrane Collaboration^®^, Oxford, England, 2020), and the SAS macro MetaDAS v1.3 [23].

## Results

### Literature search and study selection

**Fig. 1:**
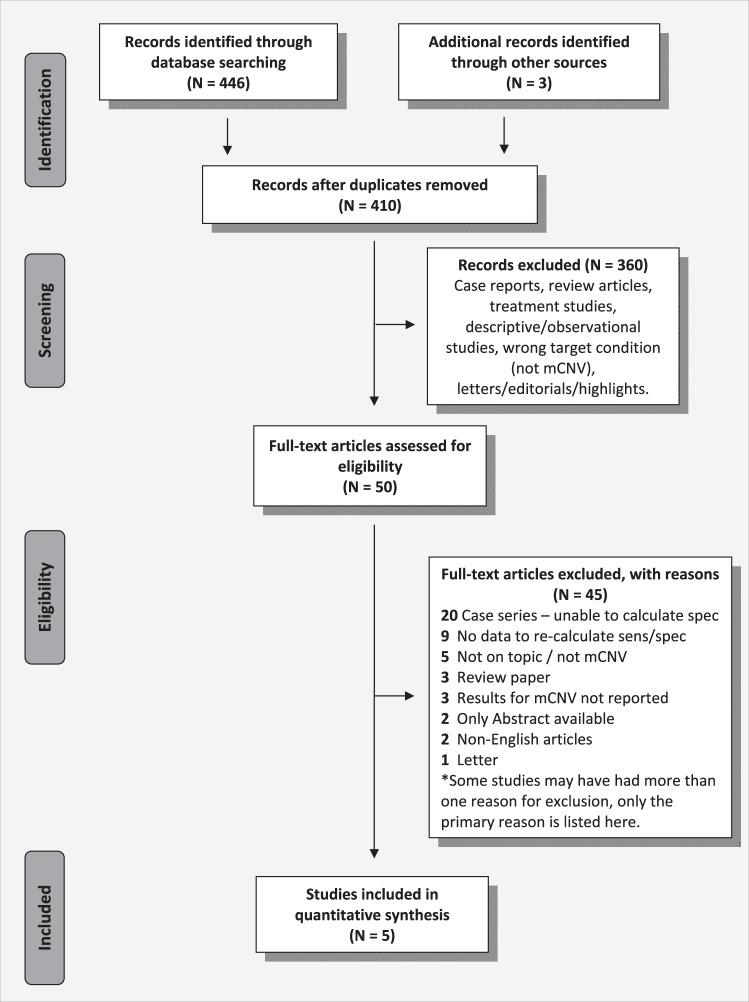
Flow diagram of the study selection process. N Number, mCNV Myopic choroidal neovascularisation, FA Fluorescein angiography, Sens Sensitivity, Spec Specificity.

The primary literature search identified 410 abstracts of potentially eligible studies. Review of retrieved abstracts and hand-searching of reference lists resulted in 50 articles for full text evaluation, of which five were deemed eligible for inclusion [24–28]. Figure 1 reports the study selection flow. Supplement eTable 2 provides additional details regarding studies deemed ineligible.

Amongst the five studies identified as being on-topic, two evaluated OCTA [25, 28], two evaluated spectral domain (SD) OCT [24, 27], and one conducted evaluations of OCTA and SD-OCT [26]. The three studies assessing test accuracy of OCTA reported complete data on a total of 95 participants (101 eyes) [25, 26, 28], whilst the three studies evaluating SD-OCT reported complete data on 130 participants (161 eyes) [24, 26, 27]. Mean (standard deviation) age was 55.90 (7.71) years in the OCTA studies and 53.91 (11.10) years in the SD-OCT studies. Additional details of the studies are reported in Table 1. Supplement eTable 3 reports detailed descriptions of the index and reference test assessment procedures.

**Table 1:** Key details of included studies.

Study	Description	Major exclusions	Participants	Sensitivity/Specificity
Bagchi et al. 2019 [26]	Retrospective audit of patients who presented to a retinal clinic in the United Kingdom with high myopia (<−6D or AL > 26 mm) and new onset visual disturbance who received FA, OCTA and SD-OCT imaging.	Excluded patients who did not receive all three imaging modalities. Excluded patients with poor quality images. Excluded patients with other co-existing major ocular conditions.	27 eyes of 26 patients (18 female, 6 male) Mean age 47.7 ± 19.7 years	OCTA vs FA: Sensitivity 19/23, specificity 3/4 SD-OCT vs FA: Sensitivity 23/23, specificity 0/4
Milani et al. 2016 [24]	Retrospective audit of patients seen at a research hospital in Italy with recent vision deterioration, pathologic myopia (<−6D and staphyloma) and suspected mCNV who received near infrared, autofluorescence, FA and SD-OCT imaging at first presentation.	Excluded patients who did not receive all four imaging modalities. Excluded patients with poor quality images. Excluded patients who had previous vitreoretinal surgery, diabetes, signs of age-related macular degeneration, or vitreoretinal interface-related pathologies.	65 eyes of 62 patients (44 female, 21 male) Mean age 66.72 years, range 18–89 Mean refraction −9.72D, range −6 to −22	SD-OCT vs FA: Sensitivity 48/49, specificity 16/16
Miyata et al. 2016 [25]	Prospective study of consecutive patients who presented to a university ophthalmology clinic in Japan with pathologic myopia (<−6D or AL > 26 mm, plus chorioretinal abnormalities) and treatment naïve exudative lesions.	Patients with OCTA images of insufficient quality were excluded from analysis.	28 eyes of 26 patients (22 female, 4 male) *Included in analysis:* 21 eyes of 20 patients (17 female, 3 male) Mean age 63.0 ± 13.6 years	OCTA vs FA: Sensitivity 16/17, specificity 4/4
Querques et al. 2017 [28]	Retrospective audit of patients who presented to a university hospital’s retinal clinic in Italy with pathologic myopia (<−8D or AL > 26.5 mm, plus characteristic degenerative changes of the sclera/choroid/retina) who were diagnosed with mCNV using FA.^a^ An additional cohort of patients with pathologic myopia and no evidence of mCNV were enrolled as a negative control group.	Excluded patients with co-existing retinal conditions, history of ocular inflammation in the study eye, significant media opacities, or large haemorrhage. Patients with OCTA images of insufficient quality or who did not have FA performed on the same day as OCTA were excluded from analysis. *Negative control group:* Excluded patients with co-existing retinal conditions, or previous ocular treatments in the study eye.	36 eyes of 28 patients (23 female, 5 male) *Included in analysis:* 21 eyes of 17 patients (14 female, 3 male) Mean age 57.8 ± 14.5 years *Negative control group:* 32 eyes of 32 patients (27 female, 5 male) Mean age 56.2 ± 14.4 years, range 26–84	OCTA vs FA^a^: Sensitivity 19/21, specificity 30/32
Su et al. 2014 [27]	Prospective study of patients who presented to a macular service centre in China with high myopia (<−6D and AL > 26.5 mm) and myopic maculopathy.	Excluded patients with other retinal or choroidal diseases, or dense cataracts.	69 eyes of 42 patients (23 female, 19 male) Mean age 47.3 ± 17.3 years, range 20–79	SD-OCT vs FA: Sensitivity 16/16, specificity 53/53

*D* Dioptres, *AL* Axial length, *FA* Fluorescein angiography, *OCTA* Optical coherence tomography angiography, *SD-OCT* Spectral domain optical coherence tomography, *mCNV* Myopic choroidal neovascularisation.

^a^Use of FA as reference standard was clarified by direct communication with the corresponding author.

### Risk of bias assessment

**Fig. 2:**
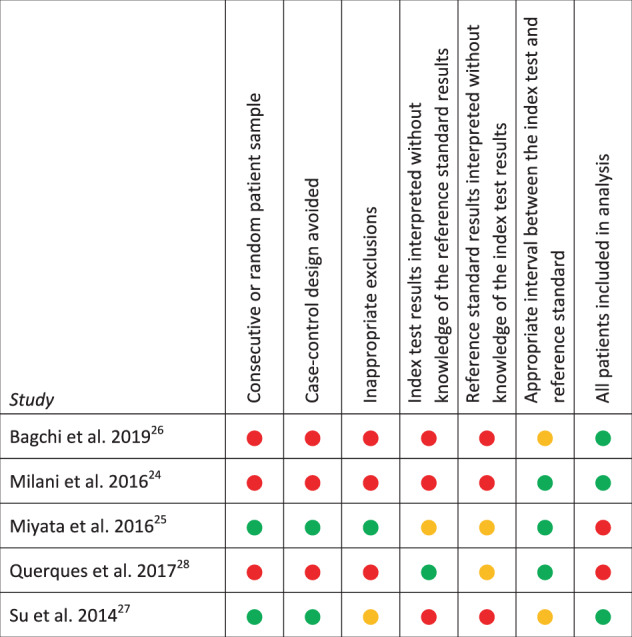
Risk of bias assessment of included studies. Low risk of bias,  Unclear risk of bias,  High risk of bias.

All five included studies were found to have at least one major methodological flaw leading to a potential high risk of bias. Two of the five studies were assessed as high risk of bias in five out of seven bias scoring domains [24, 26]. One study was assessed as high risk of bias in four out of seven bias scoring domains [28]. One study was assessed as high risk of bias in two out of seven bias scoring domains [27], whilst the remaining study was rated high risk of bias in one of the seven bias scoring domains [25]. See Fig. 2 for complete details of the risk of bias assessment.

Based on a priori defined criteria [13], publication bias could not be assessed due to the inadequate number of included studies.

### Primary analysis: test accuracy

#### Studies comparing OCTA with FA

Three studies enrolling 95 patients (101 eyes) compared the performance of OCTA against FA [25, 26, 28]. Pooled diagnostic performance showed a sensitivity of 0.89 (95% CI 0.78–0.94) and specificity of 0.93 (95% CI 0.79–0.98). The likelihood ratio of a positive test result was 11.8 (95% CI 3.96–35.25) and the likelihood ratio of a negative test result was 0.12 (95% CI 0.061–0.25).

#### Studies comparing SD-OCT with FA

Three studies enrolling 130 patients (161 eyes) compared the performance of SD-OCT against FA [24, 26, 27]. Pooled diagnostic performance showed a sensitivity of 0.99 (95% CI 0.91–1.00). The likelihood ratio of a negative test result was 0.01 (95% CI 0.001–0.095). Pooled specificity and the likelihood ratio of a positive test result were unestimatable.

Additional information such as sensitivity and specificity for individual studies, pooled positive predictive value and pooled negative predictive value for each comparison (OCTA vs FA and SD-OCT vs FA) are reported in Table 2.

**Table 2:** Test accuracy of individual studies and pooled results from meta-analysis.

					Outcome (95% CI)	Outcome (95% CI)
OCTA compared to FA						
Individual studies^a^	TP	FP	FN	TN	Sensitivity	Specificity
Bagchi 2019	19	1	4	3	0.83 (0.61–0.95)	0.75 (0.19–0.99)
Miyata 2016	16	0	1	4	0.94 (0.71–1.00)	1.00 (0.40–1.00)
Querques 2017	19	2	2	30	0.90 (0.70–0.99)	0.94 (0.79–0.99)
Pooled results from meta-analysis^b^					Sensitivity	Specificity
					0.89 (0.78–0.94)	0.93 (0.79–0.98)
					LR of a positive test	LR of a negative test
					11.8 (3.96–35.25)	0.12 (0.061–0.25)
					Positive PV	Negative PV
					0.95 (0.79–0.99)	0.85 (0.61–0.94)
SD-OCT compared to FA						
Individual studies^a^	TP	FP	FN	TN	Sensitivity	Specificity
Bagchi 2019	23	4	0	0	1.00 (0.85–1.00)	0.00 (0.00–0.60)
Milani 2016	48	0	1	16	0.98 (0.89–1.00)	1.00 (0.79–1.00)
Su 2014	16	0	0	53	1.00 (0.79–1.00)	1.00 (0.93–1.00)
Pooled results from meta-analysis^b^					Sensitivity	Specificity
					0.99 (0.91–1.00)	unestimatable
					LR of a positive test	LR of a negative test
					unestimatable	0.01 (0.001–0.095)
					Positive PV	Negative PV
					unestimatable	unestimatable

*CI* confidence interval, *OCTA* optical coherence tomography angiography, *FA* fluorescein angiography, *TP* true positive, *FP* false positive, *FN* false negative, *TN* true negative, *LR* likelihood ratio, *PV* predictive value, *SD-OCT* spectral domain optical coherence tomography.

^a^Calculated using RevMan Ver 5.4.1.

^b^Calculated using SAS macro MetaDAS v1.3.

### Sources of heterogeneity

#### Studies comparing OCTA with FA

With only three studies, a priori identified potential sources of heterogeneity could not be investigated.

#### Studies comparing SD-OCT with FA

With only three studies, a priori identified potential sources of heterogeneity could not be investigated.

To investigate whether individual study inconsistencies prevented convergence towards a pooled estimate of specificity, meta-analysis was repeated with the study by Bagchi et al. [26] removed. Reanalysis including results from the studies by Su et al. [27] and Milani et al. [24] also failed to converge on a pooled estimate of specificity; see Supplement eTable 4.

### Downstream consequences

No studies provided a head-to-head comparison of the downstream consequences (e.g. management decisions, health outcomes, resource utilisation) of using OCTA or SD-OCT to diagnose mCNV compared to using the reference standard, FA.

### Clinical recommendations

Given an overall moderate certainty of the evidence, we conditionally recommend the use of OCTA to achieve a diagnosis when mCNV is clinically suspected. With moderate confidence in the consistency of the estimate of sensitivity between studies and moderate confidence in the precision of the pooled estimate of sensitivity, we recommend OCTA as an initial test to rule out mCNV. With moderate confidence in the consistency of the estimate of specificity between studies and moderate confidence in the precision of the pooled estimate of specificity, we also recommend OCTA to rule in the presence of mCNV. However, as indicated by the wide confidence intervals around the likelihood ratios for a positive test result and a negative test result (Table 2), OCTA may have high false positive and false negative rates.

Given an overall low to moderate certainty of the evidence, we conditionally suggest clinicians may consider the use of SD-OCT to achieve a diagnosis when mCNV is clinically suspected. With high confidence in the consistency of the estimate of sensitivity between studies and high confidence in the precision of the pooled estimate of sensitivity, we recommend SD-OCT as an initial test to rule out mCNV. However, with low confidence in the consistency of the estimate of specificity between studies and an unestimatable pooled specificity, we do not recommend reliance on SD-OCT alone to rule in the presence of mCNV because the false positive rate is unknown.

Complete results of the judgements on the certainty of the evidence [11, 12] are reported in Supplement eFig. 1 and eTable 5. Clinical Guidance Recommendations are summarised in Table 3. Supplement eTable 3 reports complete eligibility criteria used by each study to identify patients who were clinically suspected to have mCNV.

**Table 3:** Key recommendations.

Test	Recommendation
OCTA	*Conditionally recommend* the use of OCTA to achieve a diagnosis when mCNV is clinically suspected. Statement was conditional because all studies excluded patients from analysis due to image quality issues. • Recommend OCTA as an initial screening study to *rule out* mCNV. • Recommend OCTA to *rule in* the presence of mCNV. However, due to a possible high false positive rate, a positive diagnosis *should* be confirmed by FA.
SD-OCT	*Conditionally suggest* clinicians may consider the use of SD-OCT to achieve a diagnosis when mCNV is clinically suspected. Statement was conditional because of the inability to estimate a pooled specificity for SD-OCT resulting in an unknown false positive rate. • Recommend SD-OCT as an initial screening study to *rule out* mCNV. • Do not recommend reliance on SD-OCT alone to *rule in* the presence of mCNV because the false positive rate of SD-OCT is unknown. A positive diagnosis *must* be confirmed by FA.
OCTA + SD-OCT	Clinicians may consider using SD-OCT if an OCTA image of sufficient quality cannot be acquired. • If either OCTA or SD-OCT return a negative result, clinicians may be fairly confident in ruling out mCNV. • If either OCTA or SD-OCT return a positive result, FA should be performed to rule out false positives and confirm the diagnosis.

*OCTA* Optical coherence tomography angiography, *mCNV* Myopic choroidal neovascularisation, *FA* Fluorescein angiography, *SD-OCT* Spectral domain optical coherence tomography.

## Discussion

This systematic review and meta-analysis investigated the test accuracy of OCTA and SD-OCT for the diagnosis of mCNV compared to FA as the reference standard. An extensive literature search identified three eligible OCTA studies and three eligible SD-OCT studies. Although meta-analysis revealed that both OCTA and SD-OCT diagnosed mCNV with high sensitivity, a pooled estimate of specificity for SD-OCT could not be calculated. These findings have implications for the clinical diagnosis of mCNV using either OCTA or SD-OCT.

Using the GRADE criteria [11, 12] to consider uncertainty, a ‘conditional recommendation’ could be made to support the use of OCTA as an initial test when mCNV is clinically suspected. This recommendation is ‘conditional’ because a large proportion of OCTA images were not of acceptable quality to achieve a positive or negative diagnosis. Studies excluded up to 25% of patients from analysis on account of image quality issues [25, 26, 28]. OCTA is known to be prone to motion and projection artefacts as many patients have poor fixation due to co-existing myopic maculopathy [4]. Future advances in imaging hardware, software tools and image processing may help overcome these image acquisition limitations [29]. Meanwhile, a weaker ‘suggestion to consider’ SD-OCT was made because pooled specificity could not be estimated. Reliance on SD-OCT alone to diagnose mCNV may result in an excessively high false positive rate leading to unnecessary over-treatment. Nevertheless, we suggest SD-OCT could be considered when an OCTA image of sufficient quality cannot be acquired.

Based on high sensitivity, if either OCTA or SD-OCT return a negative result, clinicians can be reasonably confident in ruling out mCNV. However, if either OCTA or SD-OCT return a positive result, FA should be performed to rule out false positives and confirm the diagnosis. These evidence-based guidance statements are consistent with existing expert opinions which recommend a positive diagnosis of mCNV obtained with OCTA or SD-OCT should be confirmed by FA [4, 8, 9].

The authors of the included studies reported several reasons to explain why the performance of OCTA and OCT did not match FA. For example, retinal alterations resulting from co-existing myopic maculopathy, such as lacquer cracks, retinal pigment epithelium and chorioretinal atrophy, and retinoschisis, interfered with the ability of OCTA to detect mCNV [26, 28]. The presence of submacular haemorrhage in particular may lead to false-negative detection of mCNV by OCTA compared to FA [26]. The authors further reported that poor patient fixation and projection artefacts resulted in lower quality images, thus limiting the capability of accurately detecting small or poorly perfused mCNV [28]. Finally, the small size of many mCNV lesions noted in multiple included studies [24–26] was suggested to have prohibited proper signal detection of mCNV by OCTA. SD-OCT cannot visualise fine vessels nor provide functional information on the retinal microcirculation [28], and Leveziel et al. [30] reported that the exudative features of mCNV were more obvious on FA than on SD-OCT. Milani et al. [24] reported the absence of retinal fluid, haemorrhage, and hyper-reflective foci resulted in diagnostic difficulties using SD-OCT compared to FA. SD-OCT is also prone to segmentation errors in highly myopic eyes because the thinner retina causes enhanced visualisation of choroidal vessels which may be difficult to differentiate from mCNV [25].

First described in 1961 [31], FA continues to be considered the reference standard for the diagnosis of mCNV [4]. In the studies included in this review, active mCNV was diagnosed on FA by well-defined hyperfluorescence in the early phase that increased in leakage size and intensity in the late phase [24–26, 28]. Associated blood or pigmentation showed as blocked fluorescence [26]. FA findings in inactive mCNV comprised hyperfluorescent staining of a macular scar due to fibrosis in the absence of leakage [9, 28]. Meanwhile on OCTA images, mCNV appeared as an abnormal network of bright interlacing or tangled vessels in the outer retina and choriocapillaris slabs [26, 28]. Bagchi et al. [26] additionally reported the presence of a tight vascular net and the combination of a perilesional halo with visible core were features that indicate the presence of active mCNV [26]. On SD-OCT scans, mCNV was accepted to present as a dome-shaped area of homogenous hyperreflectivity either below or above the retinal pigment epithelium [24, 26]. Other features of a positive diagnosis of mCNV on SD-OCT included an overlying fuzzy area, absence of external limiting membrane visibility, disrupted photoreceptor ellipsoid zone, presence of subretinal hyper-reflective material, and subretinal and/or intraretinal fluid [26, 28].

Methodologically rigorous test accuracy studies are necessary to inform clinical decisions regarding the provision of safe and effective patient care [32, 33]. To assess the methodological quality of the included studies, we employed a well-established, objective grading criteria developed specifically for use in systematic reviews of diagnostic tests [17]. All studies were found to contain at least one major flaw leading to an overall high risk of bias, with the most concerning potential for bias introduced by the common use (3 of 5 studies) of case-control patient sampling (Fig. 2).

Case-control studies are prone to selection bias when the cases or controls are obtained in ways such that either cases or controls are not truly representative of the broad spectrum of patients to whom the diagnostic test will be applied in practice [34]. Case-control studies are accepted to overestimate diagnostic accuracy [17, 32]. Specific to our systematic review, selection of control patients was not broad enough to allow stable estimates of false positive or true negative event rates, thus a pooled estimate of specificity for SD-OCT could not be calculated (Table 2). One remedy for this issue would be to employ population-based random or consecutive sampling with a focus on obtaining representative populations of patients who are tested but return false positive results.

Our systematic review found only 2 of 5 included studies (40%) recruited consecutive patients in order to obtain representative populations. Johnson et al. [35] evaluated the quality of diagnostic accuracy studies using OCT to diagnose glaucoma and similarly noted only 8 of 30 publications (26.7%) reported using consecutive or random selection. With both our systematic review and Johnson et al.’s systematic review identifying a need for improvements in patient selection and avoidance of case-control studies, we strongly recommend future investigators become aware of the QUADAS-2 [17] and GRADE [11, 12] risk of bias assessment and reporting criteria before starting their projects. Familiarity with these guidelines will also help to avoid other types of major methodological flaws.

### Strengths and limitations

It is unlikely our systematic review missed any relevant studies. The comprehensive literature search was performed across two major databases (MEDLINE and EMBASE) [36] using search strategies and specific terms optimised to each database (See Supplement eTable 1) [14, 15, 37]. Language restrictions were not applied to the electronic search and reference lists of retrieved articles were hand-searched for additional eligible studies. Whilst two non-English studies were identified by the search strategy [38, 39], their English-language abstracts revealed they were not on-topic and therefore would not have qualified for inclusion. Furthermore, we did not specify any test device or model restrictions in the search, which additionally increased our ability to identify meaningful studies.

Unfortunately, the comprehensive search identified relatively few small studies that were on-topic. As such, inferences could not be drawn on potential sources of heterogeneity, e.g. the patient population or specific imaging device used. Nevertheless, by using an objective risk of bias assessment tool and following established methodological guideline development processes [11, 12], we were able to communicate the uncertainty arising from lack of high-quality evidence by choosing conservative wording to frame our clinical guidance statements.

One key limitation of this study may be associated with the use of the term “choroidal neovascularisation”. Despite recommendations for standardisation towards replacing “choroidal neovascularisation” with the term “macular neovascularisation” [40], the National Library of Medicine’s MEDLINE database still uses “choroidal neovascularization” as a MeSH category to index articles on this topic. Furthermore, searching PubMed with the term “macular neovascularisation” does not map to the established MeSH category “choroidal neovascularization”. Therefore, to aid readers in finding our systematic review when conducting an electronic literature search, we used the term “choroidal neovascularisation” throughout our manuscript [16].

### Future research directions

Future studies evaluating diagnostic test performance should ensure their study population is representative of the broad spectrum of patients expected to undergo the test in practice. This is best achieved by avoiding case-series and case-control designs and enrolling consecutive or randomly selected patients [17, 41]. Furthermore, patients clinically suspected of mCNV may have co-existing diseases such as age-related macular degeneration or media opacities, thus avoiding inappropriate exclusions based on co-existing disease allows for a more representative study population and a more pragmatic estimation of test accuracy [17, 41].

Evaluations of novel diagnostic tests should be prospective and ensure the index test and reference test are applied to all enrolled patients, independent of their respective test results [17, 41]. Future projects should also aim to link clinical decisions guided by diagnostic test results to downstream patient consequences measured using validated health outcomes, such as quality of life, that adequately capture the impact of inappropriate treatment decisions [21]. Another avenue for investigation is the diagnostic accuracy of swept-source OCTA or swept-source OCT for mCNV as this newer technology allows improved visualisation of deeper retinal structures, including the choriocapillaris and choroid, due to the penetration of the longer wavelength light source and increased scanning speeds compared to SD devices [42]. Furthermore, manual segmentation of OCTA volumes has been found to increase the sensitivity for detection of choroidal neovascularisation due to various pathologies including mCNV compared to automatic segmentation, emphasising its importance in future research and test procedures [43]. Whilst this current systematic review focused on the diagnosis of mCNV, future investigators may address questions relating to the utility of OCTA/OCT for monitoring mCNV progression and resolution, and the diagnostic performance of specific imaging markers of mCNV.

We acknowledge these recommendations will require the conduct of larger studies that may need significant funding. However, appropriately conducted research will pave the way towards stronger clinical recommendations that ultimately result in practice change and improved patient outcomes.

## Conclusions

This systematic review and meta-analysis investigated the test accuracy of OCTA and SD-OCT for the diagnosis of mCNV compared to FA as the reference standard. Using the GRADE criteria to assess the certainty of the evidence, we generated a ‘conditional recommendation’ for the use of OCTA to diagnose mCNV in clinically suspected patients. However, uncertainty in the evidence supporting the use of SD-OCT to diagnose mCNV constrained us to make a ‘conditional suggestion to consider’ SD-OCT. Future large, well-conducted studies incorporating a broad spectrum of representative patients may strengthen these clinical recommendations.

## Summary

### What was known before

- mCNV is one of the most common complications of myopic macular degeneration leading to progressive central vision loss.
- Due to the time-dependent nature of treatment for mCNV, there is an urgent need to understand if innovative imaging technologies can facilitate accurate diagnosis.

### What this study adds

- To our knowledge, this is the first meta-analysis to evaluate the test accuracy of OCTA or OCT for diagnosing mCNV.
- OCTA demonstrated high sensitivity and high specificity. SD-OCT also had high sensitivity, but pooled specificity for SD-OCT could not be estimated because of limitations within individual studies.
- Clinically, OCTA can be used to reliably rule in or rule out mCNV whereas SD-OCT may only reliably rule out mCNV. A positive diagnosis obtained from either test should always be confirmed with FA.

## Supplementary information

Supplementary Information

## Data Availability

All data generated or analysed during this study are included in this published paper and its supplementary information files.
