# Supplementary material for: Diagnostic accuracy of OCTA and OCT for myopic choroidal neovascularisation: a systematic review and meta-analysis
Source: Eye (Lond). 2022 Dec 2;37(1):21–9. doi: 10.1038/s41433-022-02227-8 (PMC9829918; doi:10.1038/s41433-022-02227-8)
Supplement: Supplementary file 1 — Supplementary Information [file 41433_2022_2227_MOESM1_ESM.docx]

*Supplementary Information*

**Diagnostic accuracy of OCTA and OCT for myopic choroidal neovascularisation: A systematic review and meta-analysis**

Sharon Ho^1,2^, Angelica Ly^1,2,3^, Kyoko Ohno-Matsui^4^, Michael Kalloniatis^1,2^ and Gordon S. Doig^1,2^

^1^ Centre for Eye Health, UNSW Medicine and Health, University of New South Wales, Australia.

^2^ School of Optometry and Vision Science, UNSW Medicine and Health, University of New South Wales, Australia.

^3^ Brien Holden Vision Institute, University of New South Wales, Australia.

^4^ Department of Ophthalmology and Visual Science, Tokyo Medical and Dental University.

**Corresponding Author:**

Dr. Gordon S. Doig

Centre for Eye Health,

14 Barker Street,

Kensington NSW 2052

Australia

Email: Gordon.Doig@EvidenceBased.net

Contents

[eFigure 1. Risk of bias graphs. 3](#_Toc98606922)

[eTable 1. PubMed (MEDLINE) and EMBASE search terms. 4](#_Toc98606923)

[PubMed search 24 March 2021 4](#_Toc98606924)

[EMBASE search 30 March 2021 4](#_Toc98606925)

[eTable 2. Studies excluded after detailed review. 5](#_Toc98606926)

[eTable 3. Additional details of included studies. 10](#_Toc98606927)

[eTable 4. SD-OCT sensitivity analysis: Pooled estimates of test accuracy measures. 12](#_Toc98606928)

[eTable 5. Grading Certainty of the Evidence. 13](#_Toc98606929)

[References. 14](#_Toc98606930)

# eFigure 1. Risk of bias graphs.

Risk of bias judgments using the QUADAS-2 tool,^1^ presented as percentages across included A) OCTA studies (n=3), and B) SD-OCT studies (n=3).


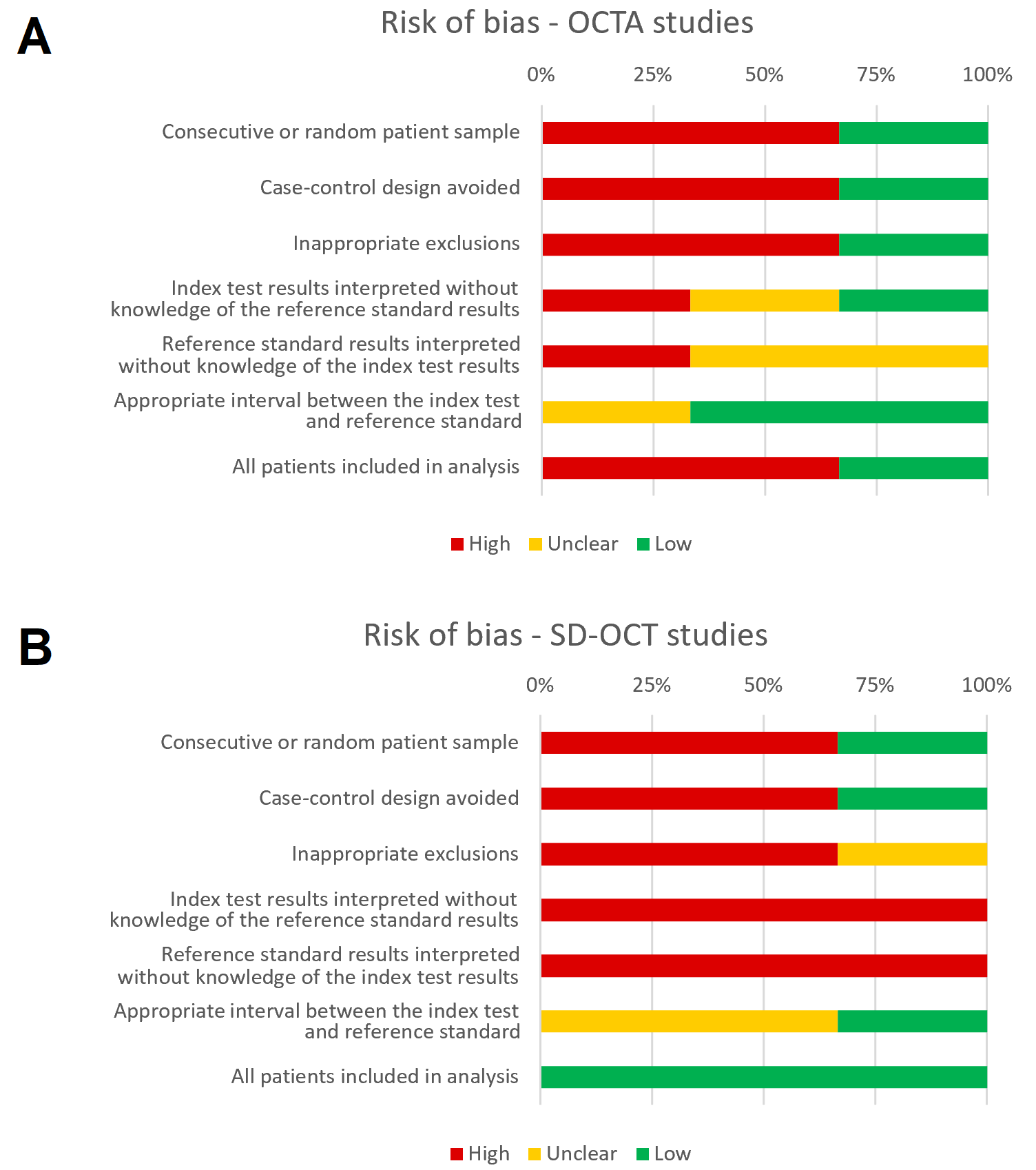


# eTable 1. PubMed (MEDLINE) and EMBASE search terms.

| PubMed search 24 March 2021 (("myopia"[MeSH Terms] OR "myopia"[All Fields] OR "myopic"[All Fields] OR "myopics"[All Fields]) AND ("choroidal neovascularisation"[All Fields] OR "choroidal neovascularization"[MeSH Terms] OR ("choroidal"[All Fields] AND "neovascularization"[All Fields]) OR "choroidal neovascularization"[All Fields])  **OR**  ("myopia"[MeSH Terms] OR "myopia"[All Fields] OR "myopic"[All Fields] OR "myopics"[All Fields]) AND ("macular degeneration"[MeSH Terms] OR ("macular"[All Fields] AND "degeneration"[All Fields]) OR "macular degeneration"[All Fields] OR "maculopathies"[All Fields] OR "maculopathy"[All Fields])  **OR**  ("myopia"[MeSH Terms] OR "myopia"[All Fields] OR "myopic"[All Fields] OR "myopics"[All Fields]) AND ("macular degeneration"[MeSH Terms] OR ("macular"[All Fields] AND "degeneration"[All Fields]) OR "macular degeneration"[All Fields])  **OR**  ("myopia"[MeSH Terms] OR "myopia"[All Fields] OR "myopic"[All Fields] OR "myopics"[All Fields]) AND "macular"[All Fields] AND ("neovascular"[All Fields] OR "neovascularisations"[All Fields] OR "neovascularities"[All Fields] OR "neovascularization, pathologic"[MeSH Terms] OR ("neovascularization"[All Fields] AND "pathologic"[All Fields]) OR "pathologic neovascularization"[All Fields] OR "neovascularisation"[All Fields] OR "neovascularity"[All Fields] OR "neovascularization"[All Fields] OR "neovascularized"[All Fields] OR "neovascularizations"[All Fields])  **OR**  "wet"[All Fields] AND ("myopia"[MeSH Terms] OR "myopia"[All Fields] OR "myopic"[All Fields] OR "myopics"[All Fields]) AND ("macular degeneration"[MeSH Terms] OR ("macular"[All Fields] AND "degeneration"[All Fields]) OR "macular degeneration"[All Fields])  **)AND(**  Clinical queries Dx filter, BROAD  (sensitiv*[Title/Abstract] OR sensitivity and specificity[MeSH Terms] OR diagnose[Title/Abstract] OR diagnosed[Title/Abstract] OR diagnoses[Title/Abstract] OR diagnosing[Title/Abstract] OR diagnosis[Title/Abstract] OR diagnostic[Title/Abstract] OR diagnosis[MeSH:noexp] OR (diagnostic equipment[MeSH:noexp] OR diagnostic errors[MeSH:noexp] OR diagnostic imaging[MeSH:noexp] OR diagnostic services[MeSH:noexp]) OR diagnosis, differential[MeSH:noexp] OR diagnosis[Subheading:noexp])  **)AND(**  "tomography, optical coherence"[MeSH Terms] OR ("tomography"[All Fields] AND "optical"[All Fields] AND "coherence"[All Fields]) OR "optical coherence tomography"[All Fields] OR ("optical"[All Fields] AND "coherence"[All Fields] AND "tomography"[All Fields]) OR (("tomography, optical coherence"[MeSH Terms] OR ("tomography"[All Fields] AND "optical"[All Fields] AND "coherence"[All Fields]) OR "optical coherence tomography"[All Fields] OR ("optical"[All Fields] AND "coherence"[All Fields] AND "tomography"[All Fields])) AND ("angiography"[MeSH Terms] OR "angiography"[All Fields] OR "angiographies"[All Fields]))) |
| --- |
| EMBASE search 30 March 2021 (myopia/ and subretinal neovascularization/  **OR**  myopia/ and macular degeneration/  )**AND**(  optical coherence tomography/  )**AND**(  di.fs OR predict:.tw OR specificity.tw) |

# eTable 2. Studies excluded after detailed review.

| **Study** | **Study details** | **Reason for exclusion** |
| --- | --- | --- |
| Avetisov 2015^2^ | Assessed fundus changes in myopia at different ocular axial lengths by means of FA and OCT.   - Article in Russian, only Abstract in English | Non-English article. |
| Baba 2002^3^ | Investigated morphological changes on OCT due to mCNV.   - Inclusion: treatment naïve mCNV documented on FA - Described characteristics of OCT findings in active/scar/atrophic stages of mCNV | Only cases were enrolled. With no control group, specificity cannot be calculated. |
| Battaglia 2016^4^ | Described morphological characteristics on OCT in active mCNV receiving anti-VEGF, and evaluated its diagnostic accuracy taking FA as reference.   - Inclusion: treatment naïve mCNV with active dye leakage on FA | Only cases were enrolled. With no control group, specificity cannot be calculated. |
| Bruyere 2017^5^ | Compared the sensitivity of OCTA versus FA and OCT in detecting mCNV.   - Inclusion: active mCNV (confirmed by both FA and OCT), treatment naïve and recurrent - OCTA detected mCNV in 18 of 20 eyes (90% sensitivity) | Only cases were enrolled. With no control group, specificity cannot be calculated. |
| Casalino 2015^6^ | Discussed imaging of mCNV by OCT.   - All hand-searched relevant references already identified | Letter to the Editor. |
| Cheung 2017^7^ | Review, guidance, and consensus statement on management of mCNV.   - Hand-searched relevant reference identified: Baba 2002^3^ | Literature review article. |
| Chhablani 2015^8^ | Assessed the intra/inter-observer agreement and diagnostic capabilities of CFP, OCT and FA for mCNV.   - Inclusion: suspected mCNV that underwent CFP, OCT and FA - Intraobserver agreement for FA and OCT was 0.54 and 0.44, respectively. Interobserver agreement between FA and OCT was 0.38 and 0.3, respectively. - Out of 34 eyes with positive diagnosis on CFP, mCNV was identified in 18 eyes on FA and 20 eyes on OCT. Diagnostic abilities of FA and OCT considering CFP as the reference standard were reported | Cannot calculate sensitivity and specificity of OCT relative to FA as reference standard. |
| Cohen 2018^9^ | Reviewed the clinical applications of OCTA.   - Hand-searched relevant references identified: De Carlo 2015^10^ and Miyata 2016^11^ | Literature review article. |
| Dansingani 2016^12^ | Described OCTA characteristics of subretinal hyper-reflective material subtypes.   - Inclusion: subretinal hyper-reflective material on OCT in the context of AMD, myopic macular degeneration, pachychoroid disease or retinal dystrophy - Myopia n=1, this eye exhibited well-defined early hyperfluorescence on FA, and flow within the lesion was detected readily by OCTA | Cannot calculate sensitivity and specificity of OCTA relative to FA as reference standard. |
| De Carlo 2015^10^ | Described the characteristics and sensitivity/specificity of OCTA for CNV detection.   - Inclusion: suspected CNV that underwent same day OCTA and FA | Results specific to mCNV not presented therefore cannot calculate appropriate sensitivity and specificity. |
| Ding 2018^13^ | Described the morphology of mCNV on OCT, before and after anti-VEGF treatment.   - Inclusion: mCNV confirmed by FA | Only cases were enrolled. With no control group, specificity cannot be calculated. |
| Elnahry 2019^14^ | Determined the prevalence of posterior segment manifestations among patients with pathological myopia, and their association with age, refractive error and axial length.   - Inclusion: pathological myopia with clear media - Diagnostic checklist of manifestations (including CNV) made for each eye - All eyes underwent OCT. FA performed in selected cases (no details on which/how many cases) | Cannot calculate sensitivity and specificity of OCT relative to FA as reference standard. |
| Fang 2019^15^ | Analysed choroidal thickness in myopic maculopathy, and subsequently established an OCT-based classification of myopic maculopathy.   - Inclusion: high myopia that underwent OCT | Data provided does not allow for calculation of sensitivity and specificity. |
| Garcia-Layana 2006^16^ | Evaluated OCT for monitoring mCNV before and after photodynamic therapy.   - Inclusion: pathological myopia and being treated with photodynamic therapy - Before treatment, FA leakage was seen in all eyes, and OCT detected the presence of either intraretinal or subretinal fluid in 32 of 33 eyes (96.96% sensitivity) | Only cases were enrolled. With no control group, specificity cannot be calculated. |
| Hong 2013^17^ | Compared a novel OCTA to FA, ICGA and CFP in imaging exudative macular disease.   - Inclusion: exudative macular disease - mCNV n=2; one showed allergic reaction to fluorescein and so did not receive FA, the other was not treatment naïve - Described and compared features of mCNV on the different modalities | Data provided does not allow for calculation of sensitivity and specificity (no primary data for mCNV detection by the modalities). |
| Iacono 2014^18^ | Evaluated the agreement between FA and OCT in detecting and monitoring mCNV activity during anti-VEGF treatment.   - Inclusion: mCNV undergoing anti-VEGF treatment | Only cases were enrolled. With no control group, specificity cannot be calculated. |
| Iacono 2021^19^ | Evaluated the agreement between FA and OCT in diagnosing and monitoring mCNV activity, and compared morphological features on OCTA to FA.   - Inclusion: active mCNV confirmed by FA | Only cases were enrolled. With no control group, specificity cannot be calculated. |
| Introini 2012^20^ | Evaluated mCNV characteristics on OCT during anti-VEGF treatment to identify which were associated with mCNV activity.   - Inclusion: recent onset active mCNV confirmed by FA | Only cases were enrolled. With no control group, specificity cannot be calculated. |
| Kang 2015^21^ | Described morphologic features of mCNV on FA and OCT, and correlated them with treatment response. | Conference proceeding, no full text. |
| Keane 2008^22^ | Described and compared OCT morphological characteristics of CNV in pathological myopia versus AMD.   - Inclusion: newly diagnosed CNV secondary to pathological myopia or AMD | Only cases were enrolled. With no control group, specificity cannot be calculated. |
| Kerimov 2008^23^ | Investigated the efficacy of Avastin anti-VEGF treatment for CNV.   - Inclusion: patients with CNV receiving Avastin treatment - Various aetiologies included: AMD n=9, mCNV n=5, idiopathic n=5 | Only cases were enrolled. With no control group, specificity cannot be calculated. |
| Kim 2016^24^ | Evaluated OCT for the assessment of mCNV activity compared to FA. | Conference proceeding, no full text. |
| Ladaique 2015^25^ | Investigated the efficacy of ranibizumab anti-VEGF treatment for mCNV, and compared the roles of OCT, FA and visual acuity in the treatment decision.   - Inclusion: active mCNV being treated with ranibizumab | Only cases were enrolled. With no control group, specificity cannot be calculated. |
| Lee 2018^26^ | Evaluated the usefulness of several OCT findings for detecting mCNV activity, using FA as reference.   - Inclusion: treatment naïve mCNV | Only cases were enrolled. With no control group, specificity cannot be calculated. |
| Lee 2020^27^ | Described clinical features of punctate inner choroidopathy.   - Inclusion: diagnosis of punctate inner choroidopathy | Did not investigate diagnosis of CNV due to myopia. |
| Leveziel 2013^28^ | Compared the contribution of FA or OCT versus both for the diagnosis of new onset mCNV.   - Inclusion: recent mCNV confirmed on at least one of either FA or OCT - Considering eyes for which data from both FA and OCT were available, diagnosis was made by FA alone in 38/62, OCT alone in 14/62, both OCT and FA in 10/62 - There was no agreement between leakage on FA and the occurrence of exudative signs on OCT | Only cases were enrolled. With no control group, specificity cannot be calculated. |
| Li 2020^29^ | Compared an OCTA diagnostic procedure for mCNV to FA.   - Inclusion: active or quiescent mCNV defined on FA and OCT | Only cases were enrolled. With no control group, specificity cannot be calculated. |
| Liu 2016^30^ | Observed CNV due to pathological myopia and idiopathy on OCTA during treatment.   - Inclusion: idiopathic CNV or mCNV confirmed by FA | Only cases were enrolled. With no control group, specificity cannot be calculated. |
| Liu 2019^31^ | Described the aetiologies and characteristics of CNV in young Chinese patients.   - Inclusion: CNV confirmed by FA - Aetiologies included pathological myopia, idiopathic, punctate inner choroidopathy, etc. | Only cases were enrolled. With no control group, specificity cannot be calculated. |
| Ma 2021^32^ | Investigated the sensitivity and specificity of multispectral imaging for detecting polypoidal choroidal vasculopathy, in a clinical setting of differential diagnosis.   - Inclusion: treatment naïve patients with symptoms, clinical findings of maculopathy, and diagnosis of central serous chorioretinopathy, AMD, polypoidal choroid vasculopathy, mCNV or idiopathic CNV - Patients underwent CFP, ICGA/FA, OCT and multispectral imaging examinations | Did not investigate diagnosis of CNV due to myopia. |
| Marchese 2019^33^ | Investigated the clinical spectrum of CNV in patients with dome-shaped macula using multimodal imaging.   - Inclusion: dome-shaped macula - All patients underwent OCT and fundus autofluorescence. Those suspected of CNV (including both mCNV and pachychoroid-associated CNV) also had OCTA and FA, and in a subset of eyes ICGA was performed - Of 30 eyes with CNV, OCTA detected CNV in 29 eyes and FA±ICGA in 27 eyes | Results specific to mCNV not presented therefore cannot calculate appropriate sensitivity and specificity. |
| Melzer 2018^34^ | Assessed the natural history and pathological changes in high myopia, and potential risk factors for the development of mCNV.   - Inclusion: high myopia at risk of developing mCNV - Three-year study recording the presence/absence of defined disease risk criteria, using CFP, and combined scanning laser ophthalmoscopy and OCT | Data provided does not allow for calculation of sensitivity and specificity. |
| Mi 2018^35^ | Assessed whether fluorescein leakage within recent subretinal haemorrhage in pathological myopia is suggestive of CNV.   - Inclusion: treatment naïve patients with one month onset of symptoms and dye leakage within subretinal haemorrhage on FA - Exudative signs on OCT observed in 22 of 25 eyes (88% sensitivity) - Abnormal vascular network on OCTA observed in 22 of 25 eyes (88% sensitivity) | Only cases were enrolled. With no control group, specificity cannot be calculated. |
| Milani 2013^36^ | Compared the sensitivity of FA alone versus FA with OCT for the diagnosis of mCNV.   - Inclusion: documentation or suspicion (based on clinical history) of mCNV, or macular exudative pathologies at FA and OCT | Cannot calculate sensitivity and specificity of OCT relative to FA as reference standard (no primary data for diagnosis by OCT alone). |
| Milani 2014^37^ | Described the features of mCNV on OCT.   - Inclusion: mCNV documented on FA, one month onset of symptoms | Only cases were enrolled. With no control group, specificity cannot be calculated. |
| Ng 2017^38^ | Summarized the clinical features, diagnosis, and use of anti‒VEGF for the treatment of mCNV.   - All hand-searched relevant references already identified | Review article. |
| Niederer 2018^39^ | Investigated risk factors for developing CNV and visual loss in punctate inner choroidopathy.   - Inclusion: diagnosis of punctate inner choroidopathy | Did not investigate diagnosis of CNV due to myopia. |
| Patel 2011^40^ | Described the clinical course of patients with punctate inner choroidopathy.   - Inclusion: punctate inner choroidopathy with CNV confirmed by FA and/or OCT | Did not investigate diagnosis of CNV due to myopia. |
| Querques 2016^41^ | Described OCTA features of mCNV and correlated them with FA and OCT findings.   - Inclusion: mCNV | Only cases were enrolled. With no control group, specificity cannot be calculated. |
| Sayanagi 2017^42^ | Described the features of myopic maculopathy on OCTA.   - Inclusion: myopic maculopathy on FA/ICGA/CFP - Described several types of myopic maculopathy (patchy atrophy, diffuse atrophy, lacquer cracks) and their associated OCTA findings. Did not investigate mCNV as a form of myopic maculopathy | Did not investigate diagnosis of CNV due to myopia. |
| Shi 2020^43^ | Compared the characteristics of choroidal transmission in punctate inner choroidopathy with or without CNV, and mCNV using OCT.   - Inclusion: myopic, acute blurred vision, evidence of hyper-reflective material on OCT - Observational study where patients underwent CFP, FA, OCT and OCTA | Data provided does not allow for calculation of sensitivity and specificity (no primary data for mCNV detection by the modalities). |
| Soomro 2018^44^ | Assessed OCTA for detecting CNV compared to multimodal imaging (FA, ICGA, OCT).   - Inclusion: treatment naïve, and suspected CNV due to AMD, polypoidal choroidal vasculopathy, chronic central serous chorioretinopathy or pathological myopia | Results specific to mCNV not presented therefore cannot calculate appropriate sensitivity and specificity. |
| Xu 2014^45^ | Described the clinical and imaging features of focal choroidal excavation complicated with CNV.   - Inclusion: focal choroidal excavation (on OCT) accompanied by CNV (on FA and OCT) - One half of the patients were emmetropic, and the others were myopic | Only cases were enrolled. With no control group, specificity cannot be calculated. |
| Yan 2018^46^ | Investigated the long-term progression pattern of myopic maculopathy.   - Inclusion: participants from the Beijing Eye study aged 40 years or older - Detection of myopic maculopathy characteristics, including mCNV, was based almost exclusively on CFP (information from the few OCT images not included in the investigation) | Data provided does not allow for calculation of sensitivity and specificity. |
| Ye 2007^47^ | Evaluated the changes in retinal function and efficacy of photodynamic therapy in patients with mCNV.   - Article in Chinese, only Abstract in English. | Non-English article. |

Abbreviations: FA, Fluorescein angiography; OCT, Optical coherence tomography; mCNV, Myopic choroidal neovascularisation; Anti-VEGF, Anti-vascular endothelial growth factor; OCTA, Optical coherence tomography angiography; CFP, Colour fundus photography; AMD, age-related macular degeneration; CNV, choroidal neovascularisation; ICGA, Indocyanine green angiography.

# eTable 3. Additional details of included studies.

| *Study* | *Details of key eligibility criteria* | *Index test details* | *Reference test details* |
| --- | --- | --- | --- |
| **Bagchi et al. 2019^48^** | **High myopia** defined as refractive error <-6D or AL >26mm.  **New onset visual disturbance** defined as blurring or scotoma.  **Clinical signs** raising suspicion of mCNV defined as grey elevated lesion or haemorrhage at the posterior pole seen on biomicroscopy.  **No details** were provided to explain whether patients who needed all three imaging modalities (OCTA, SD-OCT *and* FA) were clinically distinct from patients who were adequately diagnosed with only one or two imaging modalities. | **OCTA** (with mydriasis)   - AngioPlex Cirrus HD-OCT Model 5000 - Centred on the fovea, 3x3mm angiocube protocol: 245 B-scans repeated up to 4 times at each position, each B-scan is made up of 245 A-scans - Automated protocol displays vascular structure of choroid, choriocapillaris, deep retinal layers and superficial retinal layers - Automated segmentation adjusted manually - After correction of contrast/brightness, mCNV defined as bright interlacing or tangled group of vessels   **SD-OCT** (with mydriasis)   - Spectralis with enhanced depth imaging where possible - 3-dimensional volume set of 10x10° of macula (512 section images) - ART 7 frames/scan - mCNV defined by area of homogenous hyper-reflectivity either below or above the retinal pigment epithelium | **FA** (with mydriasis)   - HRA-2 device - mCNV defined by presence of early hyperfluorescence that increased in late frames or an area of late leakage |
| **Milani et al. 2016^49^** | **Recent vision deterioration** defined as <30 days onset with metamorphopsia and/or scotoma at the Amsler grid test.  **Pathologic myopia** defined as refractive error <-6D and staphyloma at SD-OCT.  **Suspected mCNV** defined as suspicion based on the clinical history, or macular exudative pathologies on FA and SD-OCT.  **No details** were provided to explain whether patients who needed all four imaging modalities (near infrared, autofluorescence, FA *and* SD-OCT) were clinically distinct from patients who were adequately diagnosed with less than four imaging modalities. | **SD-OCT**   - Spectralis multimodal imaging system - Multiple horizontal linear scans positioned on the centre of the macula in volume mode, ART=9 - Single horizontal and vertical scans positioned on the centre of the suspected lesion in single linear mode, ART=100 - mCNV defined by a hyper-reflective lesion   **No details** on whether mydriasis was induced. | **FA**   - Spectralis multimodal imaging system - Documentation of initial (0–45sec), middle (1–2min), and late (at least 3min) phases of the macular area after intravenous 2.5mL fluorescein - mCNV defined by initial dye uptake with hyperfluorescence and possibly late leakage |
| **Miyata et al. 2016^11^** | **Pathologic myopia** defined as spherical equivalent refractive error <-6D or AL>26mm, plus chorioretinal abnormalities such as lacquer cracks, chorioretinal atrophy and posterior staphyloma.  **Exudative lesions** defined as subretinal haemorrhage, serous retinal detachment and retinal oedema, seen on slit-lamp biomicroscopy, colour fundus photography and SD-OCT. | **OCTA** (with mydriasis)   - RTVue XR Avanti with AngioVue - Macular cube (3x3mm) protocol: 304 clusters of repeated B-scans that contained 304 A-scans each - Images obtained twice in approx. 3sec - Automatic retinal layer segmentation - Slab analysed: outer retinal level   **No details** on how mCNV was defined. | **FA** (with mydriasis)   - HRA-2 device - mCNV defined by presence of hyperfluorescence on early phase that increased in size and intensity on late phase |
| **Querques et al. 2017^50^** | **Pathologic myopia** defined as spherical equivalent refractive error <-8D or AL>26.5mm, plus characteristic degenerative changes of the sclera, choroid and retina. | **OCTA** (with mydriasis)   - AngioPlex Cirrus HD-OCT Model 5000 - Centred on the fovea, 3x3mm angio cube protocol: 245 B-scans repeated up to 4 times at each position, each B-scan is made up of 245 A-scans - Automated segmentation adjusted manually - mCNV defined as an abnormal neovascular network | **FA** (with mydriasis)   - mCNV defined by presence of well-defined hyperfluorescence on early phase with leakage in late phase, or staining of a scar   **No details** on the device used. |
| **Su et al. 2014^51^** | **High myopia** defined as spherical equivalent refractive error <-6D and AL>26.5mm.  **Myopic maculopathy** defined according to the system described by Hayashi et al.^52^ (presence of tessellated fundus, lacquer cracks, diffuse chorioretinal atrophy, patchy chorioretinal atrophy, mCNV, macular atrophy) seen on direct ophthalmoscopy. | **SD-OCT** (with mydriasis)   - Spectralis, Carl Zeiss Meditec - Both vertical and horizontal scans, axial and transverse resolutions 7 and 20µm, respectively - Automated tracking function   **No details** on how mCNV was defined on each modality. | **FA** (with mydriasis)   - Zeiss FF450 plus fundus camera |

Abbreviations: D, Dioptres; AL, Axial length; mCNV, Myopic choroidal neovascularisation; OCTA, Optical coherence tomography angiography; SD-OCT, Spectral domain optical coherence tomography; FA, Fluorescein angiography; ART, Automatic real time; HD, High definition; HRA, Heidelberg retina angiograph.

# eTable 4. SD-OCT sensitivity analysis: Pooled estimates of test accuracy measures.

| **Parameter** | **Estimate** | **Lower** | **Upper** |
| --- | --- | --- | --- |
| Sensitivity | 0.9846 | 0.8985 | 0.9978 |
| Specificity | 1.0000 | 0 | . |
| DOR | 1.422E10 | 0 | . |
| LR+ | 2.1864E8 | 0 | . |
| LR- | 0.01538 | 0.002193 | 0.1078 |

Abbreviations: DOR, Diagnostic odds ratio; LR+, Likelihood ratio of a positive test; LR-, Likelihood ratio of a negative test.

Excluded study by Bagchi et al. SAS reported “**WARNING: Standard error missing (.) for 3 estimate(s) in the table. Pooled estimates may be unstable**” and could not converge on a pooled estimate of specificity.

# eTable 5. Grading Certainty of the Evidence.

Judgements of the certainty of the evidence for included OCTA and SD-OCT studies, graded across the domains of risk of bias, indirectness, inconsistency, and imprecision.^53, 54^

| **Index test** | **Number of studies** | **Domains for grading Certainty of the Evidence** | | | | **Overall Certainty of the Evidence** |
| --- | --- | --- | --- | --- | --- | --- |
|  |  | Risk of bias | Directness to the target setting^a^ | Consistency of evidence between studies | Precision of the pooled estimate |  |
| OCTA | 3 | High | *Community:* Low  *Referral:* Moderate | *Sensitivity:* Moderate  *Specificity:* Moderate | *Sensitivity:* Moderate  *Specificity:* Moderate | Moderate |
| SD-OCT | 3 | High | *Community:* Low  *Referral:* Moderate | *Sensitivity:* High  *Specificity:* Low | *Sensitivity:* High  *Specificity:* Unestimatable | Low-Moderate |

^a^Considered the outcome of test accuracy only (i.e. excluded health outcomes). Directness was graded separately for community eye care and referral centres.

Abbreviations: OCTA, Optical coherence tomography angiography; SD-OCT, Spectral domain optical coherence tomography.

# References.

1. Whiting PF, Rutjes AW, Westwood ME, Mallett S, Deeks JJ, Reitsma JB *et al.* QUADAS-2: a revised tool for the quality assessment of diagnostic accuracy studies. *Ann Intern Med* 2011; **155**(8)**:** 529-536.

2. Avetisov SE, Budzinskaya MV, Zhabina OA, Andreeva IV, Plyukhova AA, Kobzova MV *et al.* [Fluorescein angiography and optical coherence tomography findings in central fundus of myopic patients]. *Vestn Oftalmol* 2015; **131**(4)**:** 38-48.

3. Baba T, Ohno-Matsui K, Yoshida T, Yasuzumi K, Futagami S, Tokoro T *et al.* Optical coherence tomography of choroidal neovascularization in high myopia. *Acta Ophthalmol Scand* 2002; **80**(1)**:** 82-87.

4. Battaglia Parodi M, Iacono P, Bandello F. CORRESPONDENCE OF LEAKAGE ON FLUORESCEIN ANGIOGRAPHY AND OPTICAL COHERENCE TOMOGRAPHY PARAMETERS IN DIAGNOSIS AND MONITORING OF MYOPIC CHOROIDAL NEOVASCULARIZATION TREATED WITH BEVACIZUMAB. *Retina* 2016; **36**(1)**:** 104-109.

5. Bruyère E, Miere A, Cohen SY, Martiano D, Sikorav A, Popeanga A *et al.* NEOVASCULARIZATION SECONDARY TO HIGH MYOPIA IMAGED BY OPTICAL COHERENCE TOMOGRAPHY ANGIOGRAPHY. *Retina* 2017; **37**(11)**:** 2095-2101.

6. Casalino G, Introini U, Querques G, Bandello F. Utility of the 'fuzzy area' for active myopic choroidal neovascularization detection by spectral-domain optical coherence tomography. *Ophthalmologica* 2015; **233**(1)**:** 56-57.

7. Cheung CMG, Arnold JJ, Holz FG, Park KH, Lai TYY, Larsen M *et al.* Myopic Choroidal Neovascularization: Review, Guidance, and Consensus Statement on Management. *Ophthalmology* 2017; **124**(11)**:** 1690-1711.

8. Chhablani J, Deepa MJ, Tyagi M, Narayanan R, Kozak I. Fluorescein angiography and optical coherence tomography in myopic choroidal neovascularization. *Eye (Lond)* 2015; **29**(4)**:** 519-524.

9. Cohen SY, Miere A, Nghiem-Buffet S, Fajnkuchen F, Souied EH, Mrejen S. Clinical applications of optical coherence tomography angiography: What we have learnt in the first 3 years. *Eur J Ophthalmol* 2018; **28**(5)**:** 491-502.

10. de Carlo TE, Bonini Filho MA, Chin AT, Adhi M, Ferrara D, Baumal CR *et al.* Spectral-domain optical coherence tomography angiography of choroidal neovascularization. *Ophthalmology* 2015; **122**(6)**:** 1228-1238.

11. Miyata M, Ooto S, Hata M, Yamashiro K, Tamura H, Akagi-Kurashige Y *et al.* Detection of Myopic Choroidal Neovascularization Using Optical Coherence Tomography Angiography. *Am J Ophthalmol* 2016; **165:** 108-114.

12. Dansingani KK, Tan ACS, Gilani F, Phasukkijwatana N, Novais E, Querques L *et al.* Subretinal Hyperreflective Material Imaged With Optical Coherence Tomography Angiography. *Am J Ophthalmol* 2016; **169:** 235-248.

13. Ding X, Zhan Z, Sun L, Yang Y, Li S, Zhang A *et al.* Retinal pigmental epithelium elevation and external limiting membrane interruption in myopic choroidal neovascularization: correlation with activity. *Graefes Arch Clin Exp Ophthalmol* 2018; **256**(10)**:** 1831-1837.

14. Elnahry AG, Khafagy MM, Esmat SM, Mortada HA. Prevalence and Associations of Posterior Segment Manifestations in a Cohort of Egyptian Patients with Pathological Myopia. *Curr Eye Res* 2019; **44**(9)**:** 955-962.

15. Fang Y, Du R, Nagaoka N, Yokoi T, Shinohara K, Xu X *et al.* OCT-Based Diagnostic Criteria for Different Stages of Myopic Maculopathy. *Ophthalmology* 2019; **126**(7)**:** 1018-1032.

16. García-Layana A, Salinas-Alamán A, Maldonado MJ, Sainz-Gómez C, Fernández-Hortelano A. Optical coherence tomography to monitor photodynamic therapy in pathological myopia. *Br J Ophthalmol* 2006; **90**(5)**:** 555-558.

17. Hong YJ, Miura M, Makita S, Ju MJ, Lee BH, Iwasaki T *et al.* Noninvasive investigation of deep vascular pathologies of exudative macular diseases by high-penetration optical coherence angiography. *Invest Ophthalmol Vis Sci* 2013; **54**(5)**:** 3621-3631.

18. Iacono P, Battaglia Parodi M, Papayannis A, Kontadakis S, Da Pozzo S, Cascavilla ML *et al.* Fluorescein angiography and spectral-domain optical coherence tomography for monitoring anti-VEGF therapy in myopic choroidal neovascularization. *Ophthalmic Res* 2014; **52**(1)**:** 25-31.

19. Iacono P, Giorno P, Varano M, Parravano M. Structural and optical coherence tomography angiography in myopic choroidal neovascularization: Agreement with conventional fluorescein angiography. *Eur J Ophthalmol* 2021; **31**(1)**:** 149-157.

20. Introini U, Casalino G, Querques G, Gimeno AT, Scotti F, Bandello F. Spectral-domain OCT in anti-VEGF treatment of myopic choroidal neovascularization. *Eye (Lond)* 2012; **26**(7)**:** 976-982.

21. Kang H, Kim HE, Lee JH, Lee D, Oh HS, You YS *et al.* Classification of myopic choroidal neovascularization by fluorescein angiography and optical coherence tomography. *Investigative Ophthalmology and Visual Science* 2015; **56**(7)**:** 3757.

22. Keane PA, Liakopoulos S, Chang KT, Heussen FM, Ongchin SC, Walsh AC *et al.* Comparison of the optical coherence tomographic features of choroidal neovascular membranes in pathological myopia versus age-related macular degeneration, using quantitative subanalysis. *Br J Ophthalmol* 2008; **92**(8)**:** 1081-1085.

23. Kerimov MI, Abdullayeva EA, Aliyev Kh D, Shahmaliyeva AM. Estimation of efficacy of intravitreal avastin injection for choroidal neovascularization. *Azerbaijan Medical Journal* 2008(2)**:** 60-63.

24. Kim M, Lee DH, Choi E, Byeon SH, Koh HJ, Kim SS *et al.* Optical coherence tomography findings predictive of choroidal neovascularization(CNV) activity in pathologic myopia: Correlation with fluorescein angiography in Korean patients. *Investigative Ophthalmology and Visual Science* 2016; **57**(12)**:** 2146.

25. Ladaique M, Dirani A, Ambresin A. Long-term follow-up of choroidal neovascularization in pathological myopia treated with intravitreal ranibizumab. *Klin Monbl Augenheilkd* 2015; **232**(4)**:** 542-547.

26. Lee DH, Kang HG, Lee SC, Kim M. Features of optical coherence tomography predictive of choroidal neovascularisation treatment response in pathological myopia in association with fluorescein angiography. *Br J Ophthalmol* 2018; **102**(2)**:** 238-242.

27. Lee JH, Lee SC, Park SJ, Lee CS. Punctate Inner Choroidopathy and Choroidal Neovascularization in Korean Patients. *Ocul Immunol Inflamm* 2020; **28**(1)**:** 14-19.

28. Leveziel N, Caillaux V, Bastuji-Garin S, Zmuda M, Souied EH. Angiographic and optical coherence tomography characteristics of recent myopic choroidal neovascularization. *Am J Ophthalmol* 2013; **155**(5)**:** 913-919.

29. Li S, Sun L, Zhao X, Huang S, Luo X, Zhang A *et al.* ASSESSING THE ACTIVITY OF MYOPIC CHOROIDAL NEOVASCULARIZATION: Comparison Between Optical Coherence Tomography Angiography and Dye Angiography. *Retina* 2020; **40**(9)**:** 1757-1764.

30. Liu B, Bao L, Zhang J. Optical Coherence Tomography Angiography Of Pathological Myopia Sourced and Idiopathic Choroidal Neovascularization With Follow-Up. *Medicine (Baltimore)* 2016; **95**(14)**:** e3264.

31. Liu B, Zhang X, Peng Y, Mi L, Wen F. Etiologies and Characteristics of Choroidal Neovascularization in Young Chinese Patients. *Ophthalmologica* 2019; **241**(2)**:** 73-80.

32. Ma F, Yuan M, Kozak I, Zhang Q, Chen Y. Sensitivity and Specificity of Multispectral Imaging For Polypoidal Choroidal Vasculopathy. *Retina* 2021.

33. Marchese A, Arrigo A, Sacconi R, Querques L, Prascina F, Pierro L *et al.* Spectrum of choroidal neovascularisation associated with dome-shaped macula. *Br J Ophthalmol* 2019; **103**(8)**:** 1146-1151.

34. Melzer C, Ziemssen F, Eter N, Brinkmann C, Agostini H, Haeusser-Fruh G *et al.* Design and Baseline Characteristics of the HELP Study: An Extended and Long-Term Observation of Pathological Myopia in Caucasians. *Ophthalmologica* 2018; **240**(3)**:** 167-178.

35. Mi L, Zuo C, Zhang X, Liu B, Peng Y, Wen F. Fluorescein Leakage within Recent Subretinal Hemorrhage in Pathologic Myopia: Suggestive of CNV? *J Ophthalmol* 2018; **2018:** 4707832.

36. Milani P, Massacesi A, Setaccioli M, Moschini S, Mantovani E, Ciaccia S *et al.* Sensitivity of fluorescein angiography alone or with SD-OCT for the diagnosis of myopic choroidal neovascularization. *Graefes Arch Clin Exp Ophthalmol* 2013; **251**(8)**:** 1891-1900.

37. Milani P, Pece A, Pierro L, Bergamini F. Imaging of naive myopic choroidal neovascularization by spectral-domain optical coherence tomography. *Ophthalmologica* 2014; **232**(1)**:** 28-36.

38. Ng DSC, Lai TYY, Cheung CMG, Ohno-Matsui K. Anti-Vascular Endothelial Growth Factor Therapy for Myopic Choroidal Neovascularization. *Asia Pac J Ophthalmol (Phila)* 2017; **6**(6)**:** 554-560.

39. Niederer RL, Gilbert R, Lightman SL, Tomkins-Netzer O. Risk Factors for Developing Choroidal Neovascular Membrane and Visual Loss in Punctate Inner Choroidopathy. *Ophthalmology* 2018; **125**(2)**:** 288-294.

40. Patel KH, Birnbaum AD, Tessler HH, Goldstein DA. Presentation and outcome of patients with punctate inner choroidopathy at a tertiary referral center. *Retina* 2011; **31**(7)**:** 1387-1391.

41. Querques G, Corvi F, Querques L, Souied EH, Bandello F. Optical Coherence Tomography Angiography of Choroidal Neovascularization Secondary to Pathologic Myopia. *Dev Ophthalmol* 2016; **56:** 101-106.

42. Sayanagi K, Ikuno Y, Uematsu S, Nishida K. Features of the choriocapillaris in myopic maculopathy identified by optical coherence tomography angiography. *Br J Ophthalmol* 2017; **101**(11)**:** 1524-1529.

43. Shi X, Cai Y, Luo X, Liang S, Rosenfeld PJ, Li X. Presence or absence of choroidal hyper-transmission by SD-OCT imaging distinguishes inflammatory from neovascular lesions in myopic eyes. *Graefes Arch Clin Exp Ophthalmol* 2020; **258**(4)**:** 751-758.

44. Soomro T, Talks J. The use of optical coherence tomography angiography for detecting choroidal neovascularization, compared to standard multimodal imaging. *Eye (Lond)* 2018; **32**(4)**:** 661-672.

45. Xu H, Zeng F, Shi D, Sun X, Chen X, Bai Y. Focal choroidal excavation complicated by choroidal neovascularization. *Ophthalmology* 2014; **121**(1)**:** 246-250.

46. Yan YN, Wang YX, Yang Y, Xu L, Xu J, Wang Q *et al.* Ten-Year Progression of Myopic Maculopathy: The Beijing Eye Study 2001-2011. *Ophthalmology* 2018; **125**(8)**:** 1253-1263.

47. Ye L, Lu F, Wang RS, Wang Y, Zheng B, Pan AZ *et al.* Changes of multifocal electroretinography after photodynamic therapy of choroidal neovascularization in pathological myopia. *International Journal of Ophthalmology* 2007; **7**(3)**:** 730-733.

48. Bagchi A, Schwartz R, Hykin P, Sivaprasad S. Diagnostic algorithm utilising multimodal imaging including optical coherence tomography angiography for the detection of myopic choroidal neovascularisation. *Eye (Lond)* 2019; **33**(7)**:** 1111-1118.

49. Milani P, Massacesi A, Moschini S, Setaccioli M, Bulone E, Tremolada G *et al.* Multimodal imaging and diagnosis of myopic choroidal neovascularization in Caucasians. *Clin Ophthalmol* 2016; **10:** 1749-1757.

50. Querques L, Giuffrè C, Corvi F, Zucchiatti I, Carnevali A, De Vitis LA *et al.* Optical coherence tomography angiography of myopic choroidal neovascularisation. *Br J Ophthalmol* 2017; **101**(5)**:** 609-615.

51. Su Y, Zhang X, Wu K, Ji Y, Zuo C, Li M *et al.* The noninvasive retro-mode imaging of confocal scanning laser ophthalmoscopy in myopic maculopathy: a prospective observational study. *Eye (Lond)* 2014; **28**(8)**:** 998-1003.

52. Hayashi K, Ohno-Matsui K, Shimada N, Moriyama M, Kojima A, Hayashi W *et al.* Long-term pattern of progression of myopic maculopathy: a natural history study. *Ophthalmology* 2010; **117**(8)**:** 1595-1611, 1611.e1591-1594.

53. Schünemann HJ, Mustafa RA, Brozek J, Steingart KR, Leeflang M, Murad MH *et al.* GRADE guidelines: 21 part 1. Study design, risk of bias, and indirectness in rating the certainty across a body of evidence for test accuracy. *J Clin Epidemiol* 2020; **122:** 129-141.

54. Schünemann HJ, Mustafa RA, Brozek J, Steingart KR, Leeflang M, Murad MH *et al.* GRADE guidelines: 21 part 2. Test accuracy: inconsistency, imprecision, publication bias, and other domains for rating the certainty of evidence and presenting it in evidence profiles and summary of findings tables. *J Clin Epidemiol* 2020; **122:** 142-152.
